# Annotations

**Published:** 1898-10-29

**Authors:** 


### Annotations.

### The Cold Tub.

The *Journal of Tropical Medicine* has an interesting article on the use of the cold tub in tropical countries, in which it is shown that, health-giving as it may be to those who are young and vigorous and newly arrived, it is by no means a process to be universally advised. In the tropics a daily bath of some sort becomes a necessity for purposes of cleanliness alone. But the "tub" which is indulged in by Europeans—that is immersion in cold water, or, at any rate, in water that is distinctly colder than the body for, say, five minutes—has a distinct physiological action besides the removal of dirt, and the question is whether this is good or bad. It is pointed out that the shock is relatively greater than in temperate climates, that the abstraction of heat during the process can be but ill afforded, and that the depression of temperature induced is difficult to recover from. It is also insisted on that neither the circulatory nor the respiratory organs respond to the stimulus, and that, especially in the case of those who are "getting on" in years, the digestive organs may be congested to a dangerous extent. We think that all this is very true, but that it is true for a very large number of people who live in temperate climes as well as for those whose lives are passed in the tropics. Many people who have, as they would say, been "always accustomed" to take a cold tub every morning continue the habit long after it had better have been given up. They do this partly because it is a habit, and partly because they dislike the confession of getting old which seems to be involved in giving up the customs of their more youthful days. But we are quite clear that unless good reaction very quickly follows a cold bath, and follows it without much "towelling," such tubbing is very often injurious. Whenever a man has to "rub himself warm," or when he finds that he is not right again until after his breakfast, he may feel sure that his tub is doing him harm, and that he would do better to take a warm bath, finishing off with a rapid sponge over with cold water. "For the young, vigorous, and newly-arrived youth in the tropics the cold bath may be indulged in with impunity, and it may be with benefit; but as years lapse, the tropical resident calls for bath water warmer and still warmer, until he finds that he has best health only when the water of his bath is not below the temperature of his body." And it is the same in temperate climates. On the one hand, the anæmia of the tropics renders a man more susceptible to cold; but, on the other hand, the arterial rigidity which in temperate climates is so common an associate of advancing years renders men peculiarly unable to bear that sudden displacement of blood and that sudden rearrangement of the circulation which is involved in getting into cold water.

### The Jenner Society.

At a time when the opponents of vaccination are congratulating themselves on the victory they have secured, it becomes necessary to remind the public that there is a Jenner Society, founded to keep in memory the benefits secured to the world by vaccination. Till lately the Jenner Society has been not only a modest, but a comparatively silent one; and perhaps this has been a mistake, for the anti-vaccinators have not been silent. The Jenner Society feels that it is its duty to contradict the statements of its adversaries, and has prepared a number of leaflets and pamphlets analysing the accusations of the anti-vaccinators and showing wherein they are inaccurate. They have also publications which tell what England was before vaccination was introduced, when small-pox was free to work its ravages unchecked. The reading of these would probably have a considerable effect on the opinions of many people who have had the ideas of the opponents of vaccination thrust upon them, and have never given the other side a thought—perhaps have even been prejudiced against it because it was supported by law. Unfortunately, it has not been possible for the Jenner Society to spend money as freely as the other side has done; and if it is to carry on successfully a crusade against those prejudiced persons who are so bitterly opposed to a measure whose beneficial effect has been proved by so many years of success, they must be supported much more largely than they have hitherto been. It is so decidedly everybody's business to prevent small-pox becoming again the scourge it was once that we are sure that, if the existence of this society was more generally known, it would be much more generously supported. Everyone who is interested in the question should write to the hon. secretary, Dr. Francis Bond, at Gloucester, for a list of the society's publications.

### Incorrigible Able-Bodied Paupers.

It is the boast of England that no one within her boundaries need starve, and that by aid of the Poor Laws the assumed common law right of everyone to food and shelter is effectually met; and no doubt it is because people believe that each Englishman has this right that hesitation is shown in imposing the restrictions of a jail on the inhabitants of a workhouse. Except in regard to casuals, who must do a certain tale of work before they are discharged, every workhouse inmate retains the right to discharge himself and return again to independent poverty. And all this would be well were there not, unfortunately, a class of able-bodied people who make a convenience of the Poor Law and use the workhouse as a place of rest, a refuge to which they may resort whenever a fit of idleness comes upon them. The difficulties which the class of wilful paupers set in the way of all attempts to deal leniently with the honest poor are well illustrated by a case related in the Howard Association Report for 1898—which, by the way, is rather full of good things. It is there stated that in South London in 1898 one pauper inmate was received who had earned £3 10s. in three days, and spent it in drink, &c., in the next three days, and then in the Union-house indignantly complained that his potatos were put before him unpeeled! That man, easily able to earn excellent wages, had cost the parish £550 during his successive admissions as a pauper. It seems quite clear that where able-bodied persons are received and sheltered and fed in the workhouses, the Poor Law authorities ought to be enabled to treat the cost of their relief as a debt, to be worked out in some way or other before these incorrigibles regain their liberty.

